# FGFR4 Role in Epithelial-Mesenchymal Transition and Its Therapeutic Value in Colorectal Cancer

**DOI:** 10.1371/journal.pone.0063695

**Published:** 2013-05-16

**Authors:** Alberto Peláez-García, Rodrigo Barderas, Sofía Torres, Pablo Hernández-Varas, Joaquín Teixidó, Félix Bonilla, Antonio Garcia de Herreros, J. Ignacio Casal

**Affiliations:** 1 Department of Celular and Molecular Medicine, Centro de Investigaciones Biológicas (CIB-CSIC), Madrid, Spain; 2 Chemokines and Cell Migration Laboratory, CIB-CSIC, Madrid, Spain; 3 Hospital Puerta de Hierro Majadahonda, Madrid, Spain; 4 IMIM-Hospital del Mar, Barcelona, Spain; The Chinese University of Hong Kong, Hong Kong

## Abstract

Fibroblast growth factor receptor 4 (FGFR4) is vital in early development and tissue repair. FGFR4 expression levels are very restricted in adult tissues, except in several solid tumors including colorectal cancer, which showed overexpression of FGFR4. Here, FGFR4 mutation analysis discarded the presence of activating mutations, other than Arg^388^, in different colorectal cancer cell lines and tumoral samples. Stable shRNA FGFR4-silencing in SW480 and SW48 cell lines resulted in a significant decrease in cell proliferation, adhesion, cell migration and invasion. This decrease in the tumorigenic and invasive capabilities of colorectal cancer cells was accompanied by a decrease of Snail, Twist and TGFβ gene expression levels and an increase of E-cadherin, causing a reversion to a more epithelial phenotype, in three different cell lines. In addition, FGFR4-signaling activated the oncogenic SRC, ERK1/2 and AKT pathways in colon cancer cells and promoted an increase in cell survival. The relevance of FGFR4 in tumor growth was supported by two different strategies. Kinase inhibitors abrogated FGFR4-related cell growth and signaling pathways at the same extent than FGFR4-silenced cells. Specific FGFR4-targeting using antibodies provoked a similar reduction in cell growth. Moreover, FGFR4 knock-down cells displayed a reduced capacity for *in vivo* tumor formation and angiogenesis in nude mice. Collectively, our data support a crucial role for FGFR4 in tumorigenesis, invasion and survival in colorectal cancer. In addition, FGFR4 targeting demonstrated its applicability for colorectal cancer therapy.

## Introduction

The fibroblast growth factors (FGFs) have been implicated in multiple biological processes during embryo development, wound healing, haematopoiesis and angiogenesis [1]. They bind to four FGF receptors (FGFR) designated FGFR1-4 [2]. The FGFRs structure includes a ligand-binding domain that contains three different immunoglobulin-like domains (called Ig I, Ig II and Ig III). The ligand domain is followed by a single transmembrane domain and an intracellular cytoplasmic tyrosine kinase domain. FGFR4 displays the most restricted pattern of expression to embryonic development and tissue repair [3], [4] when compared to the other three FGFRs, and its expression levels decline postnatally. In adults, FGFR4 is expressed in muscle myofibroblasts during regeneration following injury, but not in mature skeletal muscle [5]. FGF receptors dysregulation has been shown to play an important role in cancer development and progression. These alterations have been proposed to occur through overexpression, gene amplification or mutation [6].

Previously, our group identified FGFR4 as an autoantibody target in colorectal cancer (CRC) using protein microarrays [7]. In addition, we observed a clear overexpression of FGFR4 in colorectal cancer cell lines (particularly in 2 out of 4 highly metastatic colorectal cancer cell lines) with a potential association of FGFR4-expression to late stages colorectal cancer [8]. FGFR4 has been reported to be over-expressed in human breast, prostate, colon, rhabdomyosarcoma, gastric, pancreatic, hepatocellular and pituitary adenocarcinomas [4], [9], [10], [11], [12], [13], [14], [15], where it can contribute to tumor progression by multiple mechanisms [4], [9]. Moreover, FGFR4 expression levels were associated with metastatic disease and poor survival in gastric, lung, breast adenocarcinoma and rhabdomyosarcoma [16], [17], [18]. FGFR4 somatic mutations are infrequent in cancer [11], [19], [20], [21]; Arg^388^ is the most common single nucleotide polymorphism (SNP) in FGFR4, which provokes enhanced stability and prolonged activation of the receptor. It has been associated with poor prognosis for positive node breast cancer, high-grade soft-tissue sarcoma, head and neck and lung squamous cell carcinoma [9], [16], [18], [22], [23].

Among the 18 FGF ligands, FGF19 binds preferentially FGFR4 [24], although it binds also FGFR1. Binding occurs in a complex comprising heparin, FGFR4 and two FGF molecules, which triggers FGFR dimerization, leading to autophosphorylation of multiple tyrosine residues in the intracellular tyrosine kinase domain [3], [25]. FGF19-FGFR4 has been proposed to play a role in the induction of hepatocyte proliferation and carcinogenesis [26]. Antibodies directed against FGF19 have shown therapeutic promise in different tumor xenografts [27]. However, blocking of FGF19 might act on different FGF receptors [28].

We have used different colon cancer samples and cell lines (SW480, SW620, SW48, KM12C and KM12SM) [29], [30], [31] to investigate the presence of SNPs or activating mutations in FGFR4 and to characterize its biological relevance as oncogene and therapeutic target in colorectal cancer. KM12C and KM12SM epithelial cells possess similar genetic background, differing in their metastatic properties [31]. SW480 and SW620 are two isogenic colorectal cancer cell lines. SW480 was isolated from a primary Duke’s B tumor of colorectal cancer, whereas SW620 cell line was isolated from a metastatic lymph node of the same patient [30]. SW48 colorectal cancer cells was derived from a tumor at Duke’s C stage [30]. These five cell lines differ also in FGFR4 protein expression levels [8]. In our study, no new SNPs or activating mutations were found in FGFR4. However, loss-of-function experiments revealed a major role of FGFR4 in tumorigenic properties of colorectal cancer cells, since its depletion abrogated proliferation, adhesion, migration and invasion. FGFR4-silencing caused an up-regulation of E-cadherin expression and down-regulation of Snail and other epithelial-mesenchymal transition (EMT) mediators. Finally, we demonstrated that FGFR4 targeting was able to block tumor growth *in vitro* and *in vivo*.

## Materials and Methods

### Ethics Statement

The Ethical Committee of the Consejo Superior de Investigaciones Científicas (Madrid, Spain) approved the protocols used for experimental work with mice.

### Cell Lines, RNA Extraction, Antibodies and Inhibitors

Colorectal cancer cell lines KM12C and KM12SM [31], [32] were obtained from Dr I. Fidler (MD Anderson). SW480, SW48 and HEK293 cell lines were from ATCC. Cells were grown according to established protocols [7]. RNA was extracted from cell lines and 20 paired normal/tumoral tissue from cancer patients with the RNeasy Mini Kit (Qiagen Inc.) according to the manufacturer’s protocol. The extracted RNA was quantified with a NanoDrop ND-1000 spectrophotometer (NanoDrop Technologies Inc.).

A total of 15 different antibodies were used, including proteins related to the FGFR4 signaling pathway and control proteins. Source, clonality, and conditions of usage for every case and technique are specified in **Table S1**. FGFR4-specific polyclonal antibody (sc-124, Santa Cruz Biotechnology) and control GST-specific polyclonal antibody from GE Healthcare were used for FGFR4-targeting experiments. FGFR inhibitors were PD173074 (Sigma) and TKI-258 (Novartis). PD173074 is a pan-FGFR inhibitor that induces apoptosis [33]. TKI-258 is a clinically relevant, multi-kinase inhibitor, including VEGFR and PDGFR kinases among others [34]. Other inhibitors were: PP2 (Sigma) for SRC, JNK Inhibitor II and UO126 (Calbiochem) for MEK1/2. They were used at 3 µM and 15 µM as previously reported [35].

### Vectors, shRNAs, siRNAs and Transfections

pRS vectors containing specific shRNAs (TI378641, TI378642, TI378643 and TI378644) for FGFR4 (NM_022963) and a control shRNA non-effective against any human sequence (TR30003) were from Origene. Stably-transfected cells were obtained by retroviral infection. Briefly, HEK293FT cells were transfected with pRS vectors and pNGVL-gag-pol and pNGVL-VSVG packaging vectors using jetPRIME Transfection Reagent (Polyplus). After incubating the cells for 12–15 h in serum-free media, the media was replaced with DMEM containing 10% FBS and penicillin/streptomycin. The day after, media containing lentiviral particles was centrifuged, diluted 1∶2–1∶10 in DMEM containing 10% FBS and antibiotics and directly added to SW480 and SW48 colorectal cancer cells. After three days of incubation, infected SW480 and SW48 colorectal cancer cells were selected using 1 µg/ml puromycin (Sigma) for 2–3 weeks. Then, cells were cultured with 0.5 µg/ml puromycin.

FGFR4 siRNAs and controls were purchased from Sigma. For siRNA transfections, 5×10^5^ cells were seeded in culture plates and maintained in DMEM with 10% fetal calf serum at 37°C in 5% CO_2_ for 24 h. Cells were transfected with 55 pmol siRNA using 2 µl JetPrime Transfection reagent in 200 µl of JetPrime buffer. Then, 48 h after transfection, cells were analyzed by western blot and semi-quantitative PCR [35].

### Sequence Analysis, Semi-quantitative and Real-time Quantitative PCR

cDNA was synthesized using the Superscript III First Strand Synthesis kit (Invitrogen). The primers used to get the FGFR4 sequence were previously described [36]. Briefly, four pairs of primers (A, B, C and D) were used to get the whole molecule by PCR in fragments of approximately 1000 bp using the Advantage 2 polymerase (Clontech). Exonuclease I (USB) and shrimp alkaline phosphatase (USB) were added to the PCR products. They were directly sequenced in an ABI7002 sequencer (Applied Biosystems).

cDNA was synthesized as before and directly used for semi-quantitative PCR analysis of TGF-β1 and GAPDH mRNA levels in colorectal cancer cell lines. PCR reactions were performed using the following primers; human TGF-β1, sense 5′-ACCGGCCTTTCCTGCTTCTCA-3′, antisense 5′-CGCCCGGGTTATGCTGGTTGT-3′; human GAPDH, sense, 5′-GGCTGAGAACGGGAAGCTTGT-3′, antisense 5′-CGGCCATCACGCCACAGTTTC-3′. Specific primers for FGFR1-3 used to test the specificity of shRNAs and siRNAs directed against FGFR4 were: FGFR1, sense 5′-CACAAGCCACGGCGGACT-3′, antisense 5′-TGATGCTCCAGGTGGCAT-3′; FGFR2, sense 5′-CGTTGCCATTCAAGTGACTG-3′, antisense 5′- GACAAAATCTTCCGCACCATC-3′; and FGFR3, sense 5′- CAGTTGGTCTTCGGCAGC-3′, antisense 5′-TGCTGCCAAACTTGTTCT-3′.

For qRT-PCR, reactions were performed using previously described EMT marker primers [37] and SYBR-Green Master PCR mix (Applied Biosystems), in triplicate. PCR and data collection were performed on IQ5 (BioRad). All quantitations were normalized using human GAPDH. For the semi-quantitative PCR, the D pair of primers was used to determine the amount of FGFR4 cDNA, using GAPDH amplification with specific primers as control [36].

### Western Blot Analysis

Protein extracts from colorectal cancer cells were prepared and quantified with the 2D-Quant kit (GE Healthcare) according to previously published protocols [7]. Then, 25 µg of each protein extract were run in parallel using 10% SDS-PAGE. For immunoblotting, proteins were transferred to nitrocellulose membranes (Hybond-C extra) using semi-dry equipment (Bio-Rad). After blocking, membranes were incubated with specific mono- or polyclonal antibodies against the selected proteins. Membranes were incubated at optimized dilutions with primary antibodies followed by incubation with either HRP-anti-mouse IgG (Pierce) at 1∶5000 dilution or HRP-anti-rabbit IgG (Sigma) at 1∶5000 dilution. Specific reactive proteins were visualized with SuperSignal West Pico Maximum Sensitivity Substrate (Pierce). The abundance of the proteins in western blot assays was determined by densitometry using Quantity One 1D Analysis Software v4.6 (Bio-Rad Laboratories).

### Cell Adhesion, Invasion, Apoptosis Detection, Proliferation, and Wound Healing Assays

For cell adhesion assays, 96-well plates were coated with Matrigel (0.4 µg/mm^2^) (BD Biosciences) in coating buffer (0.1 M NaHCO_3_ pH: 8.8) overnight at 4°C and, then, incubated with adhesion medium (0.5% bovine serum albumin in serum-free DMEM) for 2 h at 37°C to block unspecific binding. Cells were starved without serum for 5 h and labeled with BCECF-AM (Molecular Probes) during 30 min at 37°C, detached with 2 mM EDTA in PBS and resuspended in adhesion medium. Then, 10^5^ cells were added in triplicate to plates and incubated for 30 min. To remove non-adherent cells, plates were washed twice with DMEM. Bound cells were lysed using 1% SDS in PBS and the fluorescence quantified in a Varioskan Flash Multimode Reader (Thermo Scientific). For Matrigel invasion assays, 8×10^5^ SW480 or SW48 cells were re-suspended in invasion medium (serum-free DMEM containing 0.5% BSA) and loaded onto 8 µm pore-size filters coated with 35–50 µl of 1∶3 dilution of Matrigel (BD Biosciences) in Transwells (Costar). The lower compartments of invasion chambers were filled with medium containing 10% FBS (Gibco). After 22 h of incubation at 37°C, non-invading cells were removed from the upper surface of the filter, and cells that migrated through the filter were fixed with 4% paraformaldehyde (Sigma), stained with crystal violet and the invading cells counted under a microscope. For wound healing, SW480 and SW48 cells were seeded in triplicate at a density of 10^6^ cells per well in 24-well plates. After attachment, a 1 mm-wide wound was produced in the cell monolayer, the growth medium was replaced and a picture was taken (day 0) in an Olympus CK40 microscope equipped with an Olympus DP12 camera at×40 magnification. Pictures of the same field were taken every 24 h.

For apoptosis detection assays, cells were incubated with 1 mM H_2_O_2_ for 16 h without serum. Then, cells were detached and incubated with FITC labeled-Annexin V (Miltenyi Biotec Inc.) and propidium iodide according to manufacturer’s instructions, and analyzed by cytofluorometry (Coulter Epics XL). For cell proliferation assays, experiments were carried out following established procedures [38], [39]. Briefly, the growth medium was changed 24 h after seeding (day 0) and cells were further incubated during three days. Then, medium was removed and cells were stained with 100 µl of the chromogenic dye 3-(4,5-dimethylthiazol-2-yl)-2,5-diphenyltetrazolium bromide (Sigma) at a final concentration of 1 mg/ml in DMEM. The cells were further incubated for 1 hr at 37°C and 5% CO_2_. Then, medium was carefully aspirated and 100 µl of DMSO (Sigma) were added to each well to disrupt cells. Absorbance was read at 570 nm. All the experiments were done three times in duplicate. For reduction of proliferation, cell viability was represented comparing the effect of anti-FGFR4 or anti-GST (as control) antibodies or specific inhibitors in comparison to untreated cells (100% of proliferation) incubated in the same conditions [39].

### Confocal Microscopy

Cells were fixed with 1% paraformaldehyde and permeabilised with PBS-0.5% Triton X-100 prior to the incubation with the FGFR4 specific antibody or TRITC-phalloidin for 1 h at 37°C. FGFR4 or E-cadherin antibodies were detected with AlexaFluor 488-labeled anti-rabbit IgG antibody. Cells were observed with a confocal microscope (TCS-SP5-AOBS-UV, Leica-Microsystems) after nucleus counterstaining with DAPI. Images were acquired with a 63× oil immersion objective using the Leica Confocal Software. Displayed images were captured at the same sections in the different samples.

### *In vivo* Tumor Xenografts

Swiss nude mice (Charles River) were used for metastasis and tumor xenograft studies. The Ethical Committee of the Consejo Superior de Investigaciones Científicas (Madrid, Spain) approved the protocols used for experimental work with mice.

For tumor xenografts, mice were injected subcutaneously into the right flank using 1×10^7^ SW48 stably-transfected colorectal cancer cells in 0.2 mL PBS in Matrigel diluted 1∶3. Tumor sizes were monitored at least twice a week and each animal was euthanized using a CO_2_ chamber in accordance to the Guidelines for Human Endpoints for Animal Use in Biomedical Research.

### Statistical Analysis

All statistical analyses, except where indicated, were done with Microsoft Excel. Data are presented as median ± standard deviation. For evaluation of the statistical significance compared between groups all *p* values were derived from a two-tailed statistical test with 95% confidence interval. *p* values <0.05 were considered statistically significant.

## Results

### FGFR4 Mutational Status in Colorectal Cancer Cell Lines and Tissue Samples

We investigated FGFR4 mutations in colorectal cancer cell lines and cancer samples that could contribute to antibody recognition by overexpression or activation. Total RNA was isolated and cDNA synthesized by retrotranscription. cDNA was used as template to determine the presence of mutations.

In total, we found 3 SNPs in the FGFR4 cDNA in 4 colorectal cancer cell lines and 20 cancer samples (**Table 1** and **Figure S1**). We observed three mutations in the extracellular and transmembrane domain of FGFR4 in patient samples. In addition to well-characterized Arg^388^ SNP [9], [15], we found V10L localized in the signal peptide and P136L between immunoglobulin domains 1 and 2 **(Figure S1)**. These two SNPs have been previously reported without correlation to pathological manifestations [40], [41]. In KM12C and KM12SM colorectal cancer cells, we observed the presence of the Arg^388^ SNP, whereas SW480 and SW48 did not contain this SNP. In 20 colorectal cancer samples, 60% of patients presented the polymorphism Arg^388^, whereas the prevalence of the other two mutations was 55% for P136L and 15% for V10L. These data are in agreement with previously published data, where 50% of tumors present the Arg^388^ SNP, 53% the polymorphism P136L and 30% of the tumors contain V10L [9].

**Table 1:** FGFR4 mutational status in colorectal cancer and control cell lines.

Cell line properties	Cell line	Mutation
Non metastatic	SW480	P136L
Highly-metastatic	SW48	V10L
		P136L
Low-metastatic	KM12C	V10L
		P136L
		G388R
Highly-metastatic	KM12SM	V10L
		P136L
		G388R
Pancreatic adenocarcinoma	BxPc3	P136L
Human Embrionic Kidney	HEK293	–

We did not find any activating mutation in FGFR4 or any nucleotide change out of previously reported for FGFR4. Then, these data suggest that the increased expression of FGFR4 could be responsible for autoantibody induction in CRC patients and higher invasion and metastasis in colon cancer.

### FGFR4 Knockdown Reduced Adhesion, Migration and Invasion of Colorectal Cancer Cells

**Figure 1:**
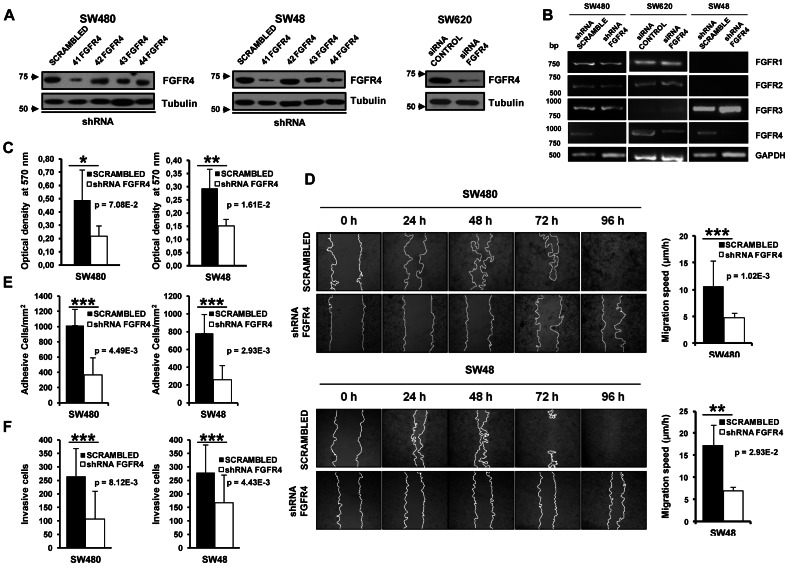
FGFR4-silencing in colorectal cancer cells decreases tumorigenic and invasive properties of colorectal cancer cells. Four shRNAs directed against different exons of FGFR4 and a scrambled shRNA were used to obtain stably transfected colorectal cancer cells after selection with puromycin. In addition, transient siRNA FGFR4-silencing was carried out in SW620 colorectal cancer cells. A. Western blot analysis of FGFR4 expression in stably transfected SW480 and SW48 cell lines, and transiently-transfected SW620 cells. Tubulin was used as loading control. B. Semi-quantitative PCR analysis of FGFR1, FGFR2, FGFR3, and FGFR4 expression using specific primers in three different cell lines. GAPDH was used as control. C. Proliferation was determined by MTT assays after 24 h of culture. Optical density was significantly decreased by FGFR4 knockdown (*, p<0.01; **, p<0.001). D. Scrambled and silenced cells were grown until confluence and their migratory capabilities were analyzed in a wound-healing assay every 24 h until confluence. Representative images of the wound-healing assay are shown. Migration speed (µm/h) of scrambled and FGFR4-silenced cells was calculated as the distance covered in 96 h. E. Cell adhesion to Matrigel of FGFR4-silenced or scrambled cells, after starving cells for 5 h in medium alone. F. SW480 and SW48 scrambled cells showed approximately 2-fold higher invasion than shRNA #41 FGFR4 stably-transfected cells. Data for all the experiments represent the mean ± SD of 3 independent experiments. *p* values of all the experiments are shown.

To study the role of FGFR4 overexpression in tumorigenesis and metastasis, we studied the effect of FGFR4 expression in SW48, SW480 and SW620 colorectal cancer cell lines, which did not contain the Arg^388^ polymorphism. SW480 and SW48 cells were stably transfected with four shRNAs targeting FGFR4 plus a control scrambled shRNA. SW620 cells were transiently silenced with siRNA. shRNA #41 showed the highest decrease in FGFR4 protein expression (**Figure 1A**) and mRNA levels (**Figure 1B**) in both cell types. shRNA #44 was also effective in reducing protein expression, particularly in SW48 cells. Similar reduction levels were obtained with transient siRNA silencing in SW620 cells. To study the effect of FGFR4 silencing on other family members, we carried out RT-PCR. mRNA levels for FGFR1, FGFR2, and FGFR3 remained unaltered after FGFR4- silencing, confirming the specificity for FGFR4 (**Fig. 1B**). Immunofluorescence analysis showed that FGFR4 expression was significantly reduced in SW480 and SW48 after transfection with shRNA 41 vectors, although some residual staining was observed in the nucleus of SW48 cells (**Figure S2**).

To assess the tumorigenic properties, we determined cell growth, adhesion, migration and invasion of FGFR4-silenced cells. Using MTT assays, FGFR4-silenced SW480 or SW48 cells showed a reduced proliferation rate when compared to scrambled cells (**Figure 1C**). To investigate the effect of FGFR4 on migration, FGFR4-silenced SW480 and SW48 cell lines were seeded in 24-well plates and analyzed by wound healing assays. FGFR4-silenced cells were unable to close the wound even after 96 h (**Figure 1D**). The retarded migration was more important for SW48 (3-fold) than SW480 cells (2-fold). These results are similar to those reported previously using two different CRC cell lines, HCT116 and HT29, and different assays [15].

In addition, using Matrigel assays, there was an important decrease in the adhesion and invasion capacity of FGFR4-silenced cells in both cell lines. SW480 cells decreased their adhesion capacity in 2.5-fold, whereas SW48 cells showed almost 4-fold reduction respect to scrambled cells (**Figure 1E**). Invasion was approximately 2-fold reduced by FGFR4 knockdown in both cell lines (**Figure 1F**). Collectively, these data support an important role of FGFR4 in tumorigenesis and invasion of colorectal cancer, being more relevant for metastatic SW48 cells.

### FGFR4 Silencing Reduced the Expression of Inducers of the Mesenchymal Phenotype

**Figure 2:**
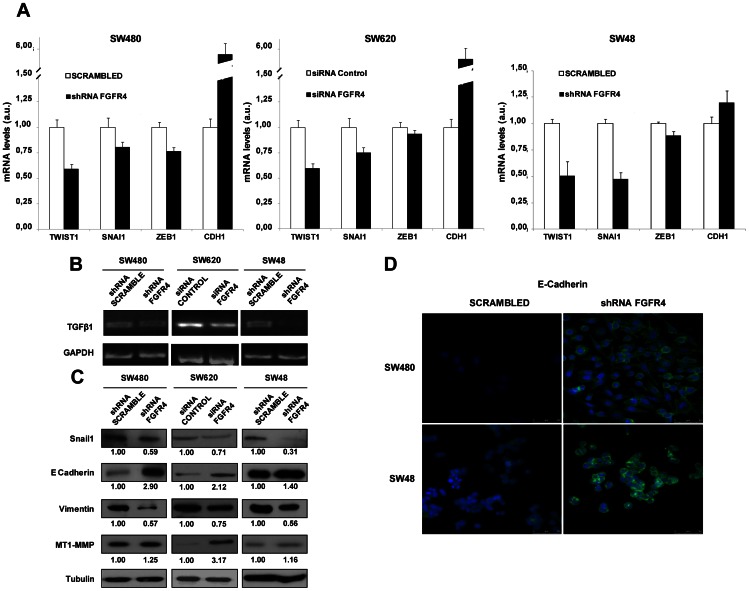
Alterations in EMT inducers after FGFR4-silencing. A. cDNA synthesized from total RNA from stably and transiently-silenced and control cells was subjected to qRT-PCR using specific primers for the EMT inducers SNAI1, TWIST1, ZEB1 and CDH1, using GAPDH for normalization. Data represent the median ± SD of two experiments. B. Same cDNA was subjected to semi-quantitative RT-PCR analysis to amplify TGFβ1, using GAPDH as control. C. SW480 and SW48 scrambled and FGFR4-silenced cells were lysed and subjected to WB analysis using specific antibodies against the indicated proteins and EMT markers. The abundance of each protein was quantified by densitometry. Tubulin was used as loading control. D. Immunofluorescence analysis of E-Cadherin in scrambled and silenced SW480 and SW48 cells. DAPI was used for counterstaining of the nucleus in blue. E-Cadherin staining was in green.

Since cell adhesion, migration and invasive capacity of epithelial cells are associated to the epithelial-mesenchymal transition (EMT), we decided to investigate alterations in EMT inducers. After FGFR4 silencing, we studied changes in mRNA expression levels of Snail1 (SNA1), TWIST1, ZEB1, and E-Cadherin (CDH1) by real-time PCR or semi-quantitative PCR (TGFβ1). In SW480 and SW620, FGFR4 knockdown caused a significant decrease in EMT inducers TGFβ1, SNA1 and TWIST accompanied by a large increase in the epithelial marker CDH1 (**Figure 2A, B**). SW48 cells exhibited a higher reduction in SNA1, TGFβ1 and TWIST1and a smaller increase in CDH1. ZEB1 decrease was more significant in SW480 than in the metastatic cell lines SW620 and SW48. Epithelial and mesenchymal markers were analyzed at the protein level by western blot. Snail and E-cadherin confirmed mRNA results. Vimentin, a mesenchymal marker, was reduced after FGFR4 silencing in the three cell lines (**Figure 2C**). Only minor changes in MT1-MMP expression were detected in SW480 and SW48 cells after FGFR4-silencing. However, we observed a large increase of MT1-MMP expression in SW620 cells, which parallels the expression levels of E-cadherin indicative of a reduction of the mesenchymal phenotype.

The increase in E-cadherin expression, after FGFR4 knockdown, in adherens junctions and cell-cell contacts was confirmed by immunofluorescence (**Figure 2D**). The differences in E-cadherin expression were more evident in the cell membrane, suggesting that FGFR4 silencing facilitated the expression of functional E-cadherin on the cell surface and the reversion to an epithelial phenotype. Collectively, these data using three different colorectal cancer cell lines confirm that FGFR4 is an important effector in EMT. FGFR4 knock-down provokes a reduction in Snail and an increase in E-cadherin with the consequent effect on adhesion, migration and invasion.

### Signaling Analysis in FGFR4-silenced Cells. Role of FGFR4 in Cell Survival

**Figure 3:**
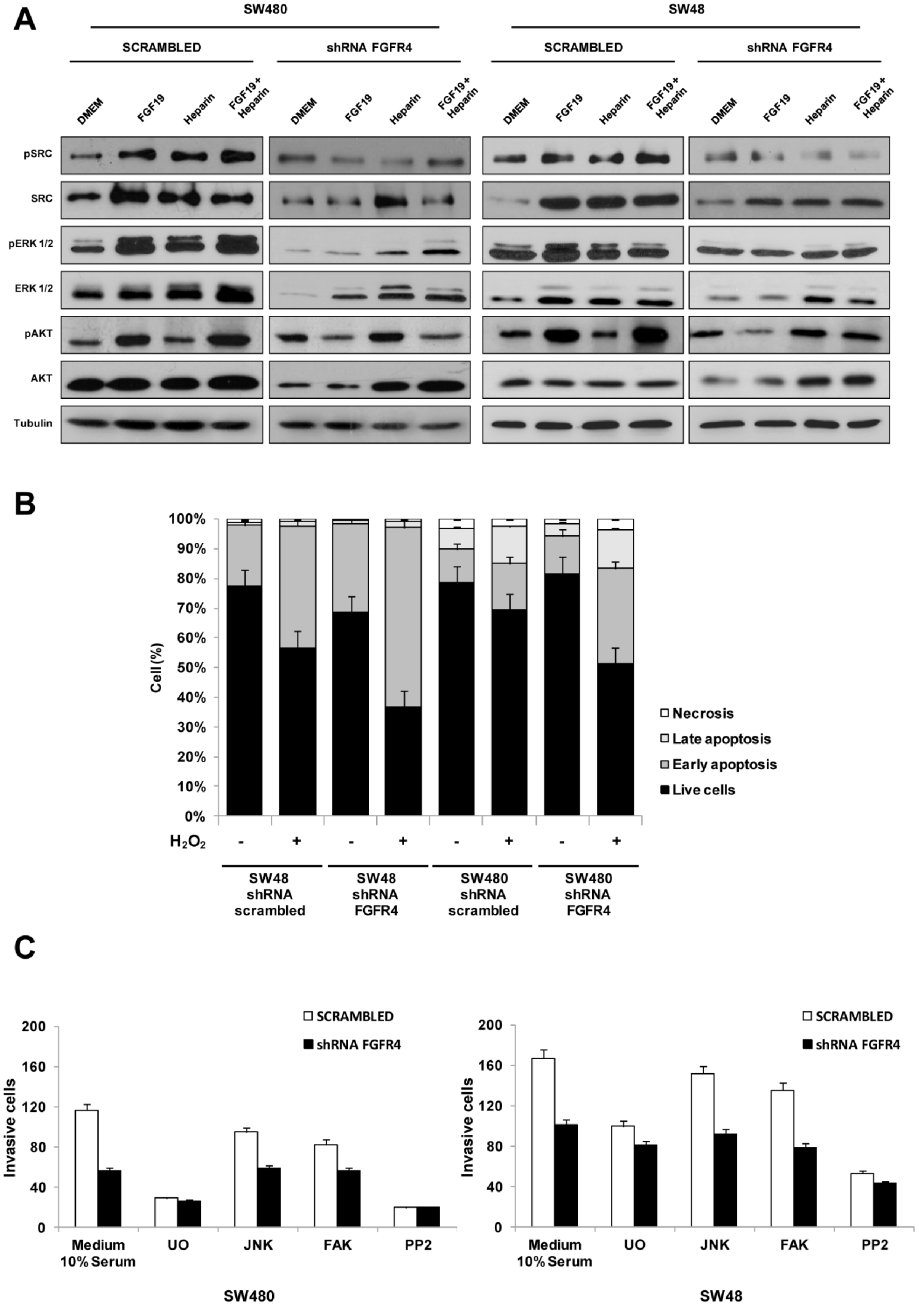
FGFR4 promotes proliferation and cell survival. A. Scrambled and FGFR4-silenced SW480 and SW48 cells were starved, and incubated with FGF19, heparin, or FGF19 plus heparin for 30 min in DMEM. Then, cells were lysed and subjected to WB analysis using specific antibodies against phosphorylated and total AKT, ERK1/2 and SRC. Tubulin was used as loading control. B. Cells were incubated in DMEM supplemented with 10% FBS and antibiotics in presence or absence of H_2_O_2_ for 16 hours, and subjected to apoptosis detection assays. Unt., untreated cells. Data showed the results for one representative experiment out of three. C. Invasion across Matrigel of scrambled and silenced cells treated with inhibitors to MEK1/2 (UO126), JNK, and SRC (PP2) as indicated. Data represent the mean ± SD of 3 independent experiments.

To determine the effect of FGFR4 activity on downstream signaling, we analyzed the effect of FGFR4-silencing on signaling pathways after activation with FGF19± heparin compared with serum-free medium. After FGFR4 knockdown, activation of phosphoSRC, phosphoERK1/2 and phosphoAKT were significantly lower in SW480 and SW48 (**Figure 3A**). ERK1 in particular showed an almost complete reduction. Regarding AKT, this effect suggests an effect mediated by FGFR4 through AKT on EMT and cell survival. To test the effect of FGFR4 on survival, cells were subjected to apoptosis induced by hydrogen peroxide. FGFR4-silenced cells showed a significant decrease of 20–30% in survival in comparison to scrambled cells after hydrogen peroxide treatment (**Figure 3B**). Without treatment, scrambled and FGFR4-silenced SW480 and SW48 colorectal cancer cells showed similar levels of apoptosis. This FGFR4 effect on cell apoptosis in response to oxidative stress may play a role in advanced colorectal cancer, facilitating the survival of metastatic colorectal cancer cells.

To study a synergistic effect to enhance FGFR4 inhibition on signaling pathways in cell invasion, we used specific inhibitors on targeted and scrambled cells. UO126 (a MEK1/2 inhibitor upstream of ERK1/2) and PP2 (SRC inhibitor) reduced the invasion capacity of SW480 and SW48 cells. This reduction was much more pronounced in scrambled than in silenced cells (**Figure 3C**). The JNK inhibitor caused only a minor effect on the invasion capacity of scrambled and silenced SW480 and SW48 cells. Collectively, these results indicate that FGFR4-knockdown caused a significant reduction of SRC and MEK1/2-ERK1/2-mediated invasion in SW480 and SW48 cells, similar to that caused by specific inhibitors on scrambled cells.

### FGFR4 as Therapeutic Target in Colorectal Cancer

**Figure 4:**
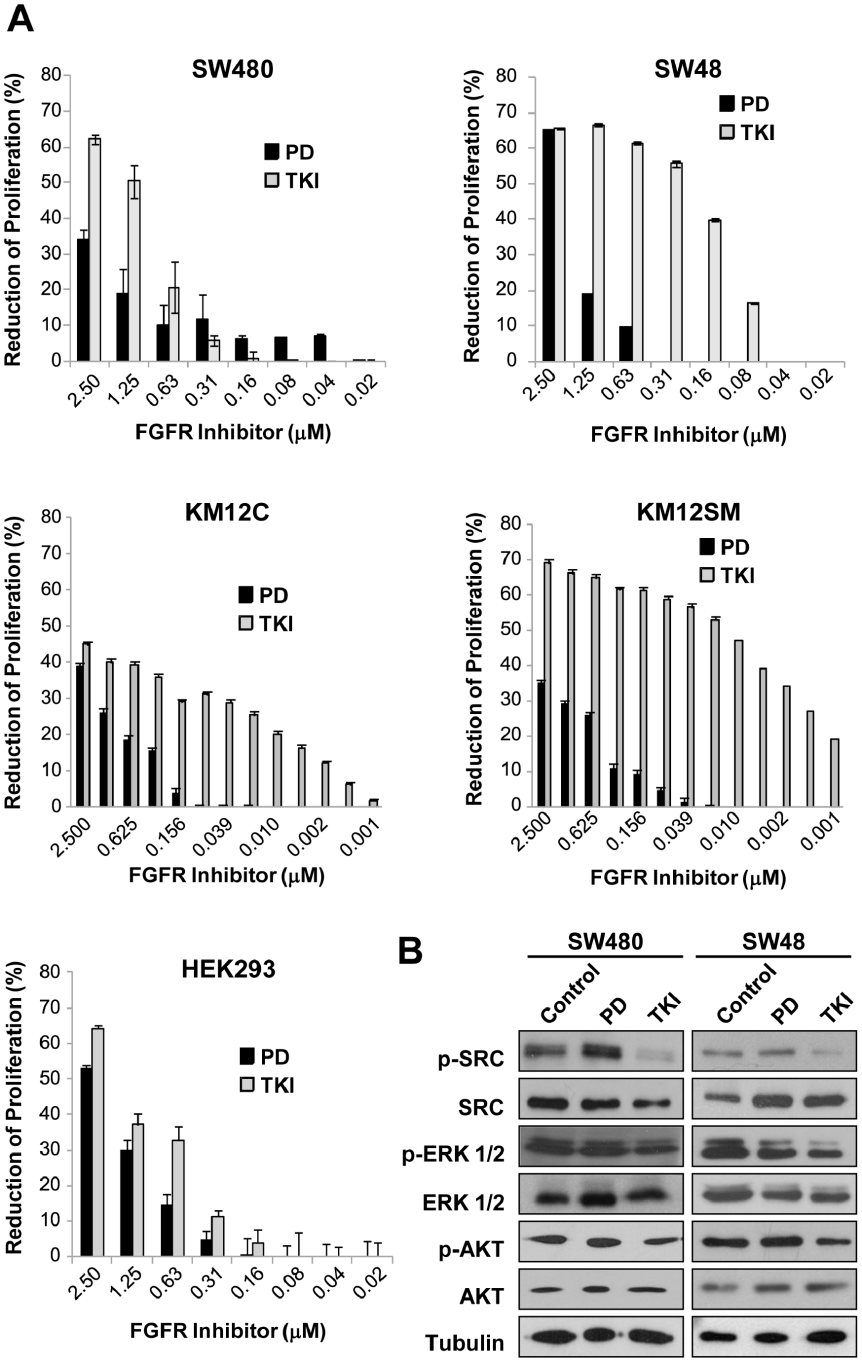
Impact of FGFR4 targeting using kinase inhibitors on colorectal cancer growth. **A.** *In vitro* cell proliferation inhibition assays were carried out using tyrosine kinase specific inhibitors. Experiments were performed in DMEM supplemented with 10% FBS and antibiotics. After 72 h of incubation with indicated concentrations, cell viability was determined by a MTT assay at 570 nm and represented as reduction of proliferation (%). Absorbance of the untreated control cells was taken as 100% of cellular growth and the reduction of the cellular growth calculated according to the following formula: (relative growth of untreated cells - relative growth of treated cells)/relative growth of untreated cells)×100. Each column is the average of three independent experiments (each concentration tested in triplicate). Error bars indicate the standard deviation of the assay. B. Targeting of FGFR4 using kinase inhibitors impairs downstream signaling pathways. Parental SW480 and SW48 cells were incubated with indicated inhibitors at a concentration of 1.25 µM for 5 h, lysed with 0.5% SDS and subjected to western blot analysis using specific antibodies against phosphorylated and total AKT, ERK1/2 and SRC. Tubulin was used as loading control.

We followed two different strategies to prove the therapeutic value of FGFR4 in colorectal cancer. First, we tested two kinase inhibitors PD173074 and TKI-258. Then, we used FGFR4-silenced cells to study the dependence on FGFR4 for tumor growth in mouse xenografts. Metastatic cell lines (SW48 and KM12SM), which express more FGFR4, were more sensitive to chemical inhibitors than poorly or non-metastatic cell lines (KM12C, SW480) (**Figure 4A**). Multi-kinase inhibitor TKI was more effective than pan-FGFR inhibitor PD to reduce colorectal cancer proliferation in a dose dependent manner (*p* value <0.001), particularly in metastatic cell lines. At 80 nM concentration, inhibition was higher in metastatic colorectal cancer cell lines (20% in SW48 and 60% in KM12SM) than in non-metastatic cells or control cells. In KM12 cells, TKI inhibition was effective up to 1 nM concentration. Reference cell line HEK293, which expresses low levels of FGFR4, was inhibited at a lower extent. Inhibition of cellular growth was associated with inhibition of same downstream regulators affected by FGFR4-signaling pathways (**Figure 4B** **)**. The most significant effects were obtained with TKI-258. We observed a vast reduction (>90%) in SRC activation, a significant decrease (50–80%) in phosphoERK1/2 and a weak reduction in phosphoAKT in both cell lines (**Figure 4B** **)**. The decrease in the activation of FGFR4-signaling pathways was similar to that observed after FGFR4-silencing, confirming the implication of FGFR4. To confirm FGFR4 specificity, we used commercial anti-FGFR4 antibodies on SW480 and SW48 colorectal cancer cells. We observed a significant inhibition of the proliferation, dose-dependent, in comparison to control cells (*p* value <0.01) (**Figure S3**). SW480 and SW48 colorectal cancer cell lines showed growth inhibition after antibody treatment, up to 75% and 60% at 400 nM and 40% and 55% at 100 nM, respectively. Effect of anti-FGFR4 antibody on control BxPc3 cells, not expressing FGFR4, was marginal at 400 nM (18%) and negligible at 100 nM.

**Figure 5:**
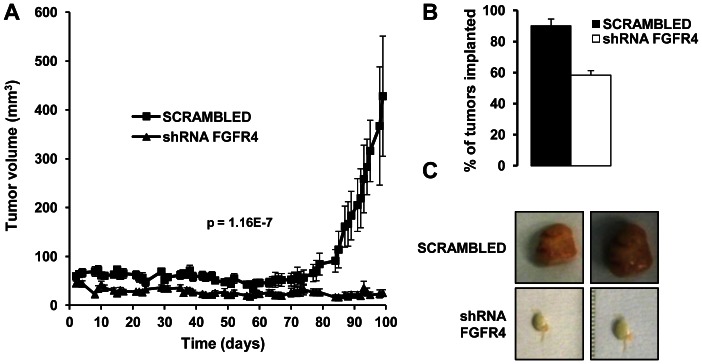
FGFR4-silencing reduces tumor growth *in vivo*. A. Scrambled and FGFR4-silenced SW48 cells were subcutaneously xenografted in swiss nude mice and the growth of the tumors formed by each cell population was followed. The statistical significance for each time point was determined by unpaired t-test. The comparison of the tumor volumes of the two mouse cohorts was statistically significant. B. Mice were visually and manually inspected to determine the number of tumors implanted. Implanted tumors were as bar graph. C. At day 100, both groups of animals were euthanized, and tumors were dissected and photographed. Dissected tumors representative for the both groups are depicted.

Finally, we injected scrambled and silenced SW480 and SW48 cells subcutaneously in nude mice to analyze the effect of FGFR4 silencing on tumor growth. Tumor development was significantly slower in mice injected with FGFR4-silenced SW48 cells (**Figure 5A**). By manual and visual inspection, we found that scrambled SW48 cells were able to form tumors in ≥90% of injected mice, whereas FGFR4-silenced SW48 cells formed tumors in ≤60% of mice (**Figure 5B**). SW480 cells did not produce tumors larger than 100 mm^3^ (data not shown). After tumor resection, there was a clear difference in the vascularization and the final tumor volume of the SW48-induced tumors (**Figure 5C**). Scrambled cells produced large spherical tumors, whereas FGFR4-silenced cells resulted in very small tumors. Moreover, their whitish aspect suggested a deficiency in angiogenesis when using silenced cells, as previously reported for FGFR1 and FGFR2 activity in glioma cells [42]. Collectively, targeting of FGFR4 with different strategies showed a great potential of this receptor as therapeutic target in colorectal cancer and a potential effect on angiogenesis.

## Discussion

FGFR targeting is becoming increasingly important for therapeutic purposes in cancer [43], [44], [45]. Among FGF receptors, FGFR1-3 have been considerably studied [46], whereas FGFR4 has been relatively less characterized at molecular level and for therapeutic purposes. Previously, we demonstrated that autoantibodies directed against FGFR4, in combination with other tumor-associated antigens (TAAs), can be used as early diagnostic markers of colorectal cancer [7], [8], [47]. Most of these TAAs were kinases with significant mutational rate (i.e PIM1, SRC, MST1 or ACVR2B among others). Although recurrent oncogenic somatic mutations have been found in FGFR2 and FGFR3 [46], only the SNP Arg^388^, present in >50% of the Caucasian ethnic group, was described in FGFR4. This polymorphism has been associated to poor prognosis and cancer aggressiveness in different cancers [4], [9], [15]. Therefore, our first goal was to investigate the presence of additional mutations in FGFR4. Sequence analysis showed the presence of the Arg^388^ polymorphism in KM12C and KM12SM colorectal cancer cells, but not in SW480, SW620 or SW48 cells. Two additional SNPs were found in colorectal cancer cell lines and patient samples, but no activating mutations were detected. No correlation was found with progression or aggressiveness. In contrast, FGFR4 in rhabdomyosarcoma showed activating mutations in 7.5% of patients [17]. Our results suggest that FGFR4 overexpression appears to be a major determinant to influence tumorigenesis and progression in colorectal cancer. Previous reports that analyzed the relevance of Arg^388^ polymorphic alleles of FGFR4 also required overexpression of FGFR4 [15].

In addition, we define a new role for FGFR4 as regulator of the epithelial-mesenchymal transition and invasion in colorectal cancer that makes it an attractive target for therapeutic intervention. Our conclusions are based on the following observations i) FGFR4 silencing caused a significant decrease in proliferation, adhesion, migration and invasion in two different cell lines, ii) pro-metastatic effects correlated with an important effect on EMT mediators like Snail, Twist or E-cadherin. FGFR4 silencing by shRNAs and siRNAs caused a recovery of epithelial markers like E-cadherin, iii) FGFR4 effects were mediated through SRC, ERK and AKT pathways, with a significant effect on cell survival, iv) silencing of FGFR4 almost abolished tumor growth in mice xenografts, and v) small molecule inhibitors and FGFR4 targeting by specific antibodies diminished cell proliferation *in vitro*. These results support the use of FGFR4 as therapeutic target for colorectal cancer. As a side conclusion, we proved that target proteins of cancer autoantibodies can be useful to identify new therapeutic targets for cancer intervention.

The EMT process is fundamental for embryonic development and involves profound phenotypic changes that include loss of cell-cell adhesion, loss of cell polarity, and the acquisition of migratory and invasive properties [48]. Association with EMT had been described for FGFR1, -2 and -3 [49], [50], [51], [52], but there were no previous data for FGFR4 implication. Particularly little was known about the relationship between FGFR activity and the transcription factors SNAIL, TWIST and ZEB1 that regulate EMT. We demonstrated that FGFR4 suppression reduces significantly the levels of TWIST in colon cancer cells, more than those of SNAIL. ZEB1 was the less affected by FGFR4 silencing. ZEB1 targets miR200 and basement proteins and seems to be more implicated in final steps of metastasis. In summary, suppression of FGFR4 expression in colorectal cancer cells produced a reversion of the mesenchymal to a more epithelial phenotype and the reduction of tumorigenic properties of colorectal cancer cells. FGFR4 regulates both the expression and the stability of TGFβ, SNAIL and TWIST genes as well as the MAPK (proliferation) and AKT (survival) pathways [25].

The relevance of FGFR4 as therapeutic target in CRC was demonstrated with multikinase inhibitors and specific antibodies, as previously done for other FGFRs [33], [44], [53]. In our hands, the promiscuous kinase inhibitor TKI-258 was much more efficient than the more selective FGFR inhibitor PD173074. It was also remarkable that KM12 cells, which contained FGFR4 Arg^388^ mutation, were extremely sensitive to TKI-258. Remarkably, drugging FGFR4 by specific inhibitors produced a reduction of key molecules of the FGFR4 signaling pathway (SRC and ERK1/2) at the same extent than FGFR4-silenced cells. This similarity of action points out that FGFR4 should be a critical target in these cells. The use of specific antibodies for FGFR4 inhibition also showed a neutralizing effect on colon cancer cells that confirmed the specificity of the inhibition. Indeed, in a recent report, it has been described that targeting FGFR4 with an specific monoclonal antibody inhibited hepatocellular carcinoma growth in a mouse xenograft model [54]. Together, these results indicate that targeting of FGFR4 might become a potent therapeutic tool in colon cancer.

In summary, we have demonstrated a potent oncogenic activity of FGFR4 in colon cancer cells. Its effect on cell migration and invasion were related to a clear regulation of EMT mediators like SNAIL, Twist or E-cadherin. The results obtained with different approaches for FGFR4 blocking indicate the efficacy of FGFR4-targeting as therapeutic alternative for colorectal cancer. It might be applied also to other cancers with high FGFR4 expression levels. We believe our findings could have direct translation into clinic by using FGFR4-based therapies.

## Supporting Information

Figure S1 FGFR4 mutational status of the CRC cell lines used in the study. A. Location of the FGFR4 mutations observed in the colorectal cancer cell lines used in this study. Protein domain boundaries were defined by the results of a search of the NCBI Conserved Domain database (NCBI CD-Search). Red, signal peptide; blue, transmembrane domain. IG, immunoglobulin-like domain; S, disulfide bond. B. Mutational status of SW480 cells. C. Mutational status of SW48 cells. D. Mutational status of KM12C cells. E. Mutational status of KM12SM cells.
(PPTX)

Figure S2 Analysis of the expression of FGFR4 by confocal microscopy with stably-transfected SW480 and SW48 cells. DAPI was used to detect the nucleus of the cells in blue. Representative micrographs show FGFR4 in green and F-actin (TRITC-phalloidin) in red.
(PPTX)

Figure S3 FGFR4 targeting using anti-FGFR4 antibodies on colorectal cancer growth. *In vitro* cell proliferation inhibition assay using FGFR4 specific antibody or an antibody against GST as control. Experiments were performed in DMEM supplemented with 10% FBS and antibiotics. After 72 h of incubation with indicated concentrations, cell viability was determined by a MTT assay at 570 nm and represented as reduction of proliferation (%). Absorbance of the untreated control cells was taken as 100% of cellular growth and the reduction of the cellular growth calculated according to the following formula: (relative growth of untreated cells - relative growth of treated cells)/relative growth of untreated cells)×100. Each column is the average of three independent experiments (each concentration tested in triplicate). Error bars indicate the standard deviation of the assay.
(PPTX)

Table S1 (XLS)
