# Supplementary material for: FGFR4 Role in Epithelial-Mesenchymal Transition and Its Therapeutic Value in Colorectal Cancer
Source: PLoS One. 2013 May 16;8(5):e63695. doi: 10.1371/journal.pone.0063695 (PMC3655941; doi:10.1371/journal.pone.0063695)
Supplement: Figure S1 — FGFR4 mutational status of the CRC cell lines used in the study. A. Location of the FGFR4 mutations observed in the colorectal cancer cell lines used in this study. Protein domain boundaries were defined by the results of a search of the NCBI Conserved Domain database (NCBI CD-Search). Red, signal peptide; blue, transmembrane domain. IG, immunoglobulin-like domain; S, disulfide bond. B. Mutational status of SW480 cells. C. Mutational status of SW48 cells. D. Mutational status of KM12C cells. E. Mutational status of KM12SM cells. (PPTX) [file pone.0063695.s001.pptx]

## Slide 1
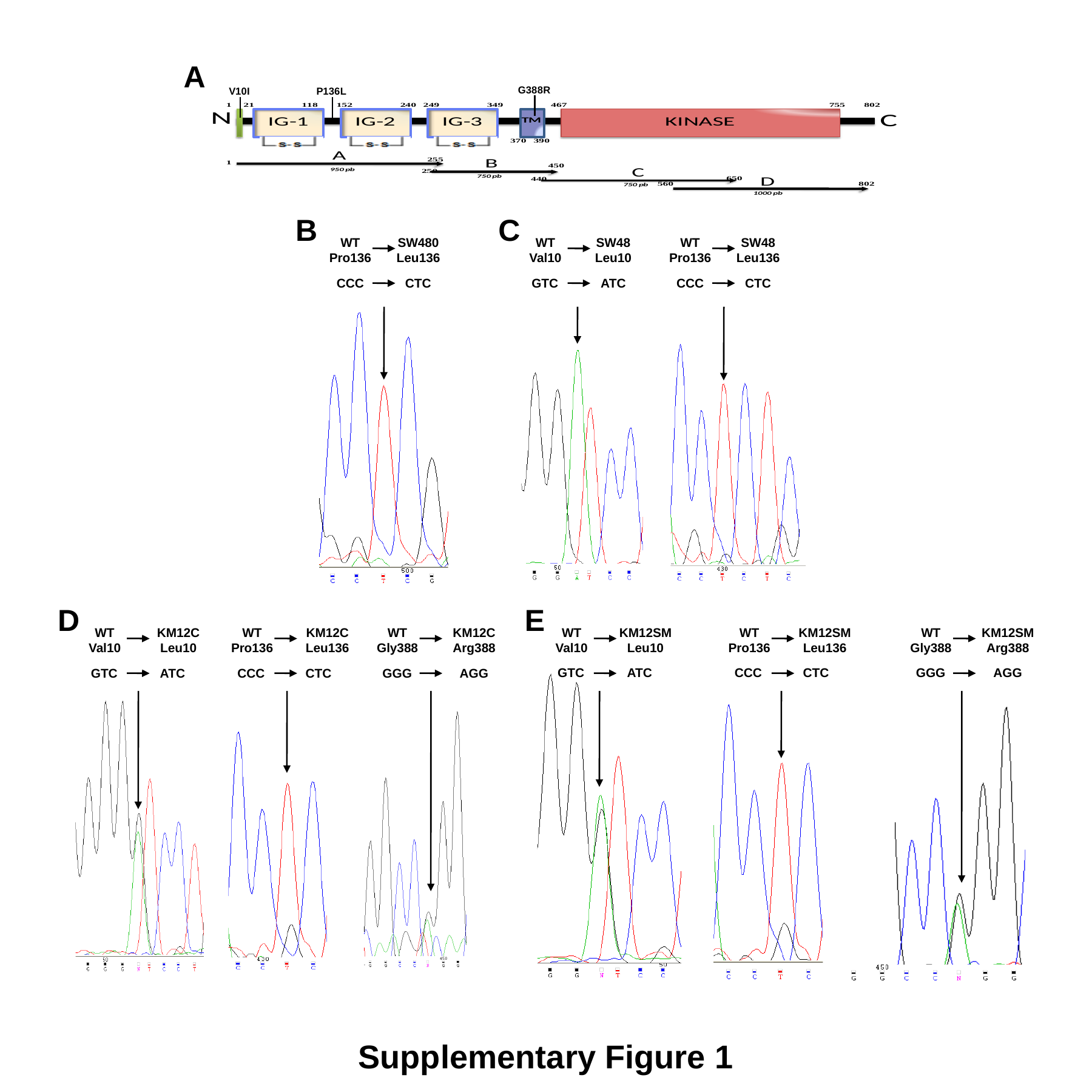

A
B
C
WT
Pro136
SW480
Leu136
WT
Val10
SW48
Leu10
WT
Pro136
SW48
Leu136
GTC
ATC
CCC
CTC
CCC
CTC
D
E
WT
Val10
KM12C
Leu10
WT
Pro136
KM12C
Leu136
WT
Gly388
KM12C
Arg388
WT
Val10
KM12SM
Leu10
WT
Pro136
KM12SM
Leu136
WT
Gly388
KM12SM
Arg388
GTC
ATC
CCC
CTC
GGG
AGG
GTC
ATC
CCC
CTC
GGG
AGG
Supplementary Figure 1
