# Supplementary figures and images for: FGFR4 Role in Epithelial-Mesenchymal Transition and Its Therapeutic Value in Colorectal Cancer
Source: PLoS One. 2013 May 16;8(5):e63695. doi: 10.1371/journal.pone.0063695 (PMC3655941; doi:10.1371/journal.pone.0063695)

## Slide 1
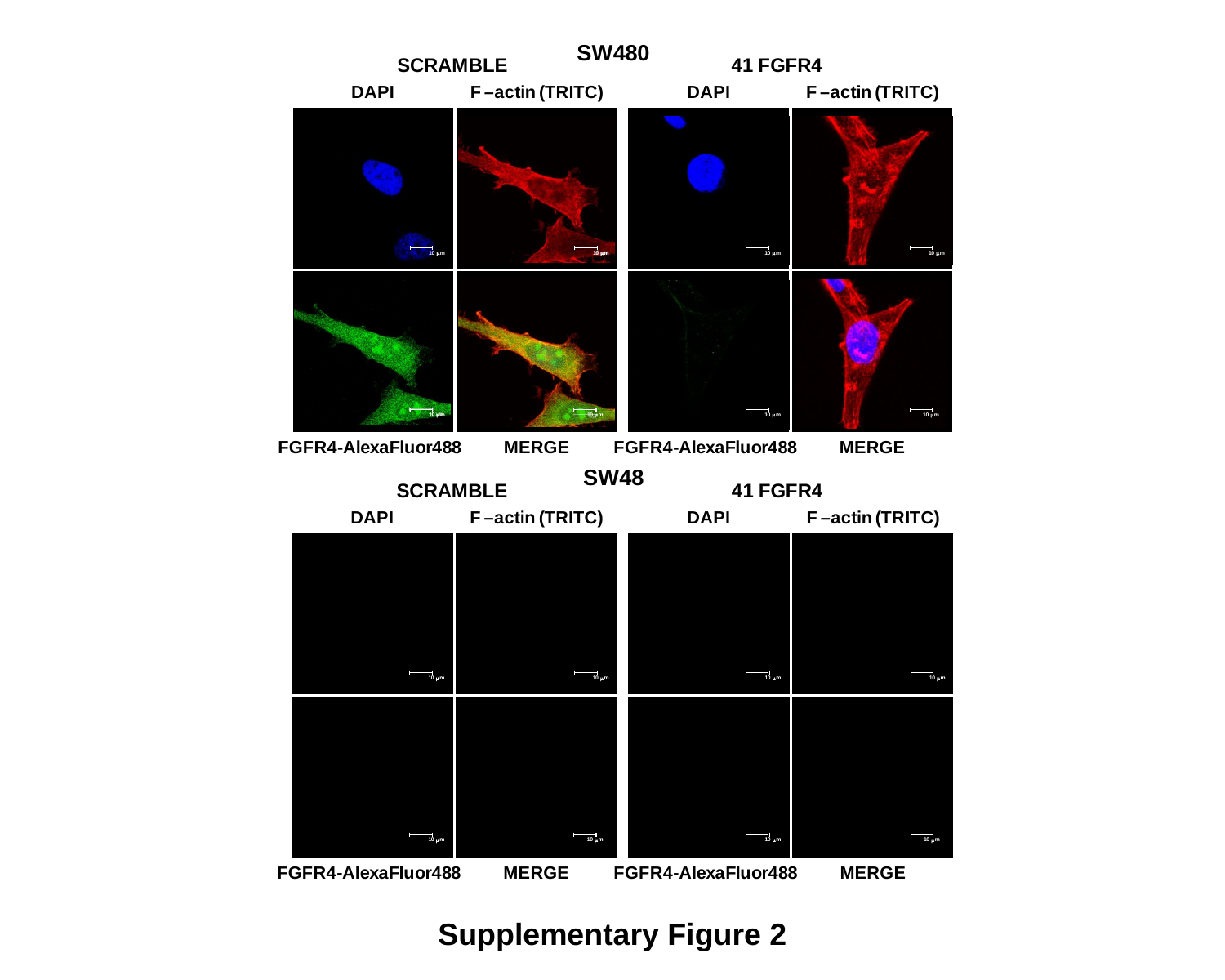

Supplementary Figure 2

Supplement: Figure S2 — Analysis of the expression of FGFR4 by confocal microscopy with stably-transfected SW480 and SW48 cells. DAPI was used to detect the nucleus of the cells in blue. Representative micrographs show FGFR4 in green and F-actin (TRITC-phalloidin) in red. (PPTX) [file pone.0063695.s002.pptx]
