# Supplementary material for: FGFR4 Role in Epithelial-Mesenchymal Transition and Its Therapeutic Value in Colorectal Cancer
Source: PLoS One. 2013 May 16;8(5):e63695. doi: 10.1371/journal.pone.0063695 (PMC3655941; doi:10.1371/journal.pone.0063695)
Supplement: Figure S3 — FGFR4 targeting using anti-FGFR4 antibodies on colorectal cancer growth. In vitro cell proliferation inhibition assay using FGFR4 specific antibody or an antibody against GST as control. Experiments were performed in DMEM supplemented with 10% FBS and antibiotics. After 72 h of incubation with indicated concentrations, cell viability was determined by a MTT assay at 570 nm and represented as reduction of proliferation (%). Absorbance of the untreated control cells was taken as 100% of cellular growth and the reduction of the cellular growth calculated according to the following formula: (relative growth of untreated cells - relative growth of treated cells)/relative growth of untreated cells)×100. Each column is the average of three independent experiments (each concentration tested in triplicate). Error bars indicate the standard deviation of the assay. (PPTX) [file pone.0063695.s003.pptx]

## Slide 1
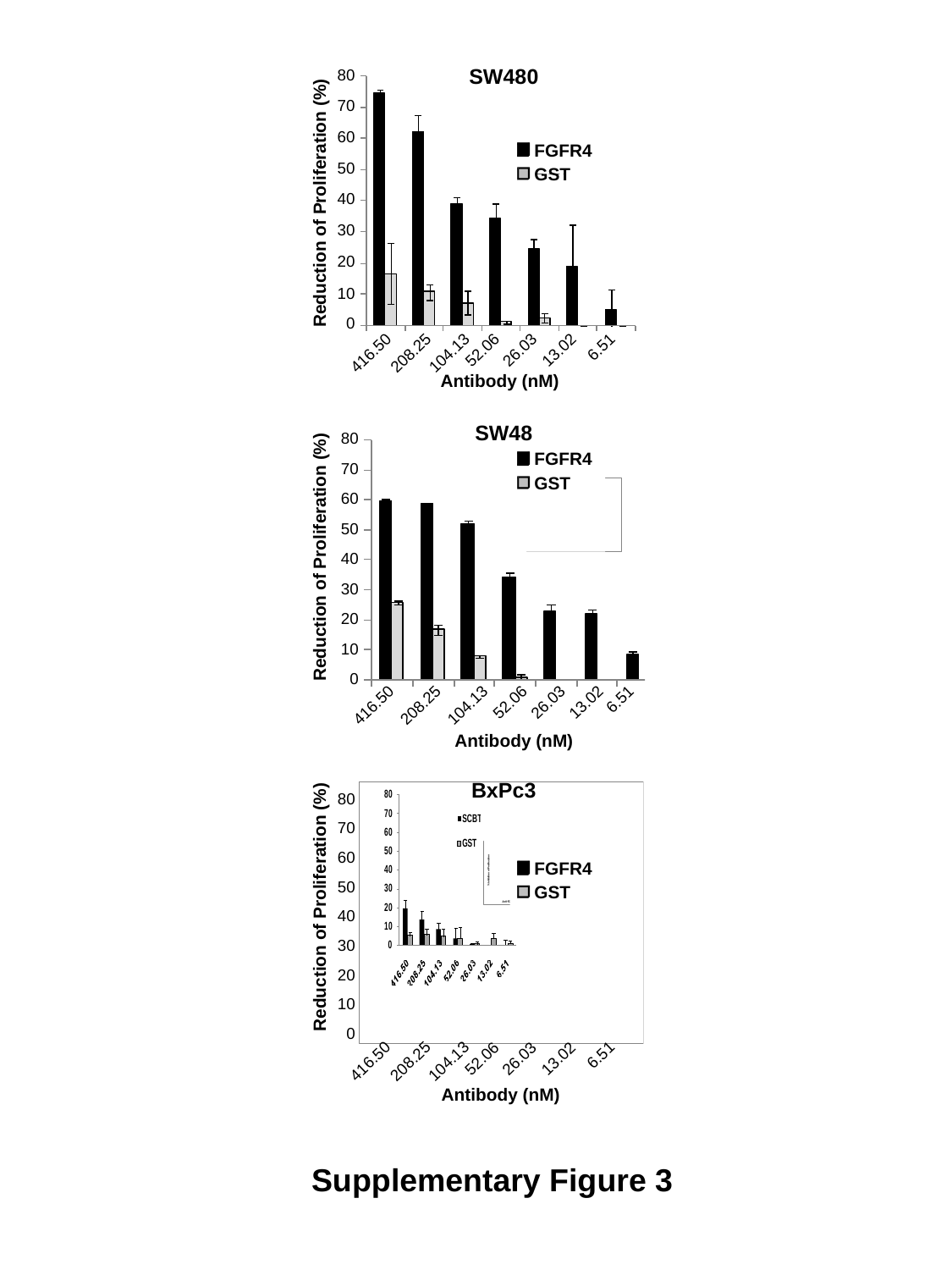

SW480
80
70
60
FGFR4
GST
50
40
Reduction of Proliferation (%)
30
20
10
0
6.51
52.06
26.03
13.02
416.50
208.25
104.13
Antibody (nM)
SW48
80
FGFR4
GST
70
60
50
Reduction of Proliferation (%)
40
30
20
10
0
6.51
52.06
26.03
13.02
416.50
208.25
104.13
Antibody (nM)
BxPc3
80
70
60
FGFR4
GST
50
Reduction of Proliferation (%)
40
30
20
10
0
6.51
52.06
26.03
13.02
416.50
208.25
104.13
Antibody (nM)
Supplementary Figure 3
